# Seasonal Variations in the Chemical Composition of Liangshan Olive Leaves and Their Antioxidant and Anticancer Activities

**DOI:** 10.3390/foods8120657

**Published:** 2019-12-08

**Authors:** Bixia Wang, Jipeng Qu, Shiling Feng, Tao Chen, Ming Yuan, Yan Huang, Jinqiu Liao, Ruiwu Yang, Chunbang Ding

**Affiliations:** 1 College of Life Sciences, Sichuan Agricultural University, Yaan 625014, China; 696wbx@163.com (B.W.); ququ8312@163.com (J.Q.); fengshilin@outlook.com (S.F.); chentao293@163.com (T.C.); yuanming@sicau.edu.cn (M.Y.); shirley11hy@163.com (Y.H.); liaojinqiu630@sicau.edu.cn (J.L.); yrwu@sicau.edu.cn (R.Y.); 2 College of Environmental Science and Engineering, China West Normal University, Nanchong 637009, China; 3 College of Agricultural Science, Xichang University, Xichang 615000, China

**Keywords:** Liangshan olive leaves, chemical composition, phenolic extracts, anticancer activity

## Abstract

The seasonal changes in the chemical composition of *Olea europaea* leaves from January to December at Liangshan (China) have been investigated. The highest total phenolic content (TPC), total flavonoid content (TFC), and free amino acid content (FAAC) levels were found in May and December, while the lowest levels were detected in April and September. The soluble protein content (SPC) and the soluble sugar content (SSC) were highest in spring but lowest in summer and winter. The levels of major phenolic compounds, including oleuropein, and luteolin-4’-O-glucoside, followed by apigenin-7-O-glucoside, quercetin, rutin, luteolin, and apigenin, increased during spring and winter but decreased during summer and autumn. In addition, phenolic extracts (PEs) showed dose-dependent antioxidant activity using 2,2-diphenyl-1-picrylhydrazyl (DPPH) radical and superoxide radical scavenging activity assays; the reducing power was tested. The anticancer activities of PE at various concentrations were assessed by a cell counting kit-8 (CCK-8), and the IC_50_ (50% effective concentration) to HEK293, HeLa, and S180 cells were 841.48, 7139, and 457.69 μg/mL, respectively. PE-treated S180 cells inhibited proliferation through activation of caspase-3/9 and disruption of the mitochondrial membrane potential. Thus, PE in Liangshan olive leaves possessed strong antioxidant and anticancer potential, and spring and winter were determined as optimal harvesting seasons.

## 1. Introduction

Olive trees (*Olea europaea* L.), belonging to the family Oleaceae, are one of the important economic crops all over the world [1]. In 1960, olive trees were introduced from the Mediterranean region into China as a commercial crop [2,3]. According to statistical analysis, almost 80,000 hectares of olive trees were cultivated by the end of 2017, generating approximately 600,000 tons/year of abandoned leaves in China [4,5]. Olive leaves, an agricultural by-product obtained after the pruning and harvesting of olive trees, are thrown away, burned, or scattered in the field, potentially causing environmental damage and increasing waste disposal cost for farmers [5,6]. Olive leaves were highly valued in Mediterranean folk medicine for the treatment of influenza, common cold, malaria, dengue, diarrhea, and surgical infections [3,7]. Because of their health promoting properties, olive leaves have recently gained increasing interest and have been used as an inexpensive raw material for various technological, scientific, and commercial applications [1,8,9]. 

Recent studies have demonstrated that olive leaves are mainly composed of moisture, proteins, lipids, minerals, and carbohydrates [10]. Although olive leaves contain large quantities of nutrients, the phenolic content is of major interest because of its health benefits [11]. Olive leaves contain an abundance of high-quality polyphenols. These compounds are mostly classified into secoiridoids, acids, and flavonoids, and they exhibit strong preventive effects against oxidation [12,13]. Based on the potential health benefits, several studies have evaluated the effect of phenolic extracts (PEs) derived from olive leaves in the treatment of various diseases, such as cardiovascular diseases, cancer, myocardial oxidative damage, and atherosclerosis [13,14,15]. In particular, oleuropein, the main phenol in olive leaves, exhibits remarkable biological and pharmacological activities, especially antioxidant, antimicrobial, and anticancer effects [16,17,18].

Seasonal variation in chemical compositions is a well-known phenomenon in plants, and it is associated with the biosynthesis, stability, and degradation of secondary metabolites in olives [19,20]. In addition, quantitative and qualitative changes in the biochemical composition of olive leaves also depends on the plant variety, climatic conditions, sampling time, genetics, and geographical origin [19,21,22]. Recent studies have typically focused on olives grown in a few countries in the Mediterranean region. In China, the Liangshan variety is produced on the largest scale under climate conditions characterized by four different seasons and variations in “weather within 10 km” [23,24]. The climatic conditions in Liangshan strongly affect the genetic quality of olive cultivars, olive fruit, and oil [24,25,26]. For example, Chen et al. [24] reported that olives from China, which possess a higher moisture content in the fruit, show unique characteristics compared with olives grown in the Mediterranean region. However, data related to the bioactive ingredients of olive leaves at different times under the climate of Liangshan are available. Determination of the seasonal effects on the bioactive constituents in Liangshan olive leaves is essential for understanding the impact of harvesting time on olive leaves and ensuring optimal concentrations of active ingredients.

The aim of this study was to investigate the seasonal variations in the chemical compositions of Liangshan olive leaves from January to December, including total phenolic content (TPC), total flavonoid content (TFC), free amino acid content (FAAC), soluble sugar content (SSC), and soluble protein content (SPC), as well as the contents of seven major phenolic compounds. In addition, the in vitro antioxidant capacities of PE as scavengers of 2,2-diphenyl-1-picrylhydrazyl (DPPH) and superoxide anions, as well as the reducing power, were evaluated. The potential anticancer effects of PE on human embryo kidney cells (HEK293), human cervical cancer cells (Hela), and ascites tumor cells (S180) were also assessed.

## 2. Material and Methods

### 2.1. Materials and Chemicals

Trees of *O. europaea* cultivar “Manzanillo” are major olive cultivars grown in Liangshan, Sichuan (China). The leaves were sampled on the 15th of each month from January to December in 2018. After collection, the leaves were dried and ground. Then, the dried powder was sieved and stored at −20 °C.

The following standards were purchased from Acros Organics (Geel, Belgium) and Chengdu Kelong Chemical Factory (Chengdu, China): methanol and acetonitrile, 2,2-diphenyl-picrylhydrazyl (DPPH), and ethanol. All reagents were of analytical grade, while six phenolic compounds were high-performance liquid chromatography (HPLC) grade (Chengdu Must-biotechnology, Chengdu, China).

### 2.2. Sample Preparation 

Powdered olive leaves (10 g) were mixed in ethanol–water (1:1) solution in a sample-to-extract ratio of 1:25 (*v*/*w*). The beaker containing the mixture was placed in an ultrasonic device (KQ300DV, 40 kHz, Kunshan Ultrasonic Instrument Co. Jiangsu, China) at 60 °C, and the extraction was performed at 270 W for 40 min. The crude extracts were centrifuged at 4500 rpm for 10 min. Subsequently, the supernatants were filtered using 0.45 µm syringe filters and were used directly to estimate TPC, TFC, and FAAC, as well as to perform HPLC analyses of the phenolic compounds.

The sample solution obtained in January was concentrated with a rotary evaporator and dried in a vacuum drying oven to obtain PE. The PE samples were stored at −20 °C in the dark for further analysis.

### 2.3. Determination of Chemical Composition

#### 2.3.1. Total Phenolic Content (TPC)

The amount of TPC in the olive leaves was estimated according to the Folin–Ciocalteu method [27]. Briefly, the extract (0.1 mL) was reacted with 2.5 mL of Folin–Ciocalteu reagent for 2 min, followed by the addition of 1 mL sodium carbonate (7.5% *w*/*v*), after which it was diluted to 10 mL with distilled water. The tube containing the reaction solution was incubated at 25 °C for 90 min without light. The absorbance was measured by a microplate reader (Spectramax M2, USA) at 760 nm. The TPC was expressed as milligrams of gallic acid equivalent (GAE) per gram dry weight of the sample (mg GAE/g).

#### 2.3.2. Total Flavonoid Content (TFC)

Total flavonoid content (TFC) of the extract was estimated by the AlCl_3_ (aluminum chloride) method with some modifications [28]. Briefly, a 2 mL sample was added to 4 mL AlCl_3_ (1% *w*/*v*), and the solution was adjusted to 25 mL with distilled water. The absorbance was measured at 415 nm. The TFC was expressed as milligrams of routine equivalent (RE) per gram dry weight of the sample (mg RE/g).

#### 2.3.3. Free Amino Acid Content (FAAC)

The FAAC of the olive leaves was determined using ninhydrin colorimetry with slight modifications [29]. Briefly, the sample supernatant (0.5 mL) was mixed with 1.5 mL ninhydrin (2% *w*/*v*), 0.5 mL distilled water, and followed by the addition of 0.05 mL ascorbic acid (0.1% *w*/*v*). Finally, the mixture was incubated at 100 °C for 10 min and cooled immediately in an ice-water bath for 20 min. The absorbance was measured at 570 nm. The FAAC was expressed in terms of glutamate standard.

#### 2.3.4. Soluble Sugar Content (SSC)

The SSC of the olive leaves was determined based on the method of Dubois et al. [30]. The method entailed mixing 0.1 g of dried powder in 5 mL of distilled water. The sample tube was immersed in an 80 °C water bath. After 30 min, 0.2 mL solution was reacted with a mixture of 0.2 mL sample solution (6% *w*/*w*) and 0.5 mL sulfuric acid. The absorbance at 490 nm was recorded, with glucose solution as the standard.

#### 2.3.5. Soluble Protein Content (SPC)

The SPC of the olive leaves was determined based on the method of Meda et al. [31]. A lysis buffer consisting of 10 mM NaCO_3_ and 10 mM NaHCO_3_ was prepared. The Coomassie Brilliant Blue (CBB) color reagent was obtained by mixing 5 mL ethanol (95%) and phosphoric acid (85%) and diluting to 100 mL. The olive dried powder (0.25 g) was extracted using lysis buffer (5 mL) in an ultrasonic device (240 W) at 20 °C for 50 min. The extracts were centrifuged (8000 rpm, 5 min), and 0.1 mL supernatant was distilled to 0.3 mL with lysis buffer. Finally, 0.5 mL CBB color reagent was added. After 2 min, the absorbance was read at 595 nm. SPC was used to quantify the standard curve with bovine serum albumin (BSA) as the reference.

### 2.4. HPLC Analysis of Phenolic Compounds

Analyses of phenolic compounds were identified and quantified on an Agilent 1260 high-performance liquid chromatography (HPLC) chromatogram (Agilent Technologies Agilent Technologies Singapore International Pte., Ltd.Singapore). The sample was separated by a Agilent 1260 HPLC system equipped with a UV–vis DAD detector at 350 nm, and detected on a Zorbax Eclipe Plus-C18 column (5.0 µm, 150 × 4.6 mm). The injection volume was 10 µL, and the temperature was maintained at 30 °C. The mobile phase comprised solvent A (0.2% aqueous phosphoric acid) and solvent B (acetonitrile). The flow rate was 0.8 mL/min, and the gradient profile was 84% A to 16% B at 0–3 min, 70% A to 30% B at 3–20 min, 60% A to 40% B at 20–25 min, 84% A to 16% B at 25–30 min, and 84% A to 16% B at 30–33 min. The amounts of the phenolic compounds were calculated using the calibration curve (C), and the equation was as follows: extraction yield (mg/g) = [(C × V)/W]/1000, where V is the volume of the extract (mL), and W is the dried weight of sample (g).

The oleuropein was determined at 254 nm during 10 min. The mobile phase A was water (75%), and mobile phase B was acetonitrile (25%).

### 2.5. Antioxidant Activity Assays

#### 2.5.1. Superoxide Radical-Scavenging Activity

The superoxide radical-scavenging activity of PE was determined according to the method of Jia et al. [32]. Briefly, PE was dissolved in deionized water and diluted with different concentrations of PE (0.2–2.0 mg/mL) to obtain sample solution. Then, the sample solution was mixed 0.2 mL nitroblue tetrazolium (NBT, 0.08 mM), 0.4 mL nicotinamide adenine dinucleotide (NADH, 0.25 mM), and 0.2 mL phenazine methyl sulfate (PMS, 0.06 mM) to obtain reaction solution. The reaction mixture was incubated at 25 °C for 15 min. The deionized water and ascorbic acid (Vc) were used as the blank and positive control, respectively. The absorbance at 560 was measured. Inhibition of scavenging effect was calculated with the following equation: scavenging activity (%) = (1 − (A_s1_ / A_s2_) / A_s0_) × 100, where A_s0_, A_s1_, and A_s2_ are the absorbance of the control, the reaction solution, and the sample solution, respectively.

The IC50 (50% effective concentration) values were calculated from the dose–response curve using SPSS 19, where the abscissa represented the concentration of tested PE as the average inhibition percentage.

#### 2.5.2. DPPH Radical-Scavenging Activity (DPPH)

The DPPH radical-scavenging activity was assayed based on the method of Brand-Williams et al. [33]. Briefly, different solutions of PE (0.2–1.2 mg/mL) were reacted with DPPH solution (0.4 mM) in ethanol. The reaction solution was incubated in the dark at 37 °C for 10 min, and the absorbance was measured at 517 nm. Ethanol served as the blank control, and Vc served as the positive control. Inhibition of scavenging activity was calculated by the equation: scavenging activity (%) = (1 − (A_s1_/A_s2_)/A_s0_) × 100, where A_s0_, A_s1_, and A_s2_ are the absorbance of the control, the reaction solution, and the sample solution, respectively.

#### 2.5.3. Reducing Power

The reducing power was evaluated as described in the methods of Moein et al. [34]. Briefly, 0.5 mL phosphate buffer (0.2 M, pH 6.6) was mixed various concentrations of PE (0.2–1.2 mg/mL) and 0.5 mL potassium ferricyanide (1% *w*/*v*) for 20 min at 50 °C. A total of 0.5 mL trichloroacetic acid (10% *w*/*v*) was supplemented and centrifuged (3000 rpm, 10 min). The 0.5 mL supernatant was made up with 0.5 mL distilled water. The mixture reacted with 0.1 mL ferric chloride (0.1% *w*/*v*). Distilled water served as the blank control, and Vc served as the positive control. Then, the absorbance at 700 nm was read. 

### 2.6. Anticancer Activity Assays

#### 2.6.1. Cell Culture

HEK293, HeLa, and S180 cells were kindly provided by the cell storeroom of the Chinese Academy of Sciences (Shanghai, China). Cells were grown in Dulbecco’s modified Eagle’s medium (DMEM) containing 10% fetal bovine serum (FBS) and 1% penicillin–streptomycin. Culture plates were placed in an incubator maintained at 37 °C with 5% CO_2_. 

#### 2.6.2. Cell Viability 

Cytotoxicity and cell viability assays were analyzed by the CCK-8 assay, as detailed by the manufacturing company (Dojindo Molecular Technologies, Tokyo, Japan). HEK293, HeLa, and S180 cells were plated in 96-well plates at an initial density of 2 × 10^4^ cells/mL. Cells were added with different concentrations of PE and were cultured for different times. After different times, the old medium of DMEM was completely removed, and 100 µL fresh growth medium was supplemented. After 2 h of incubation (37 °C, 5% CO_2_), the density of each well was recorded at 450 nm. Inhibition rate was determined using the following formula: (Ac − As)/(Ac − Ab) × 100%, where As, Ac, and Ab are the absorbance of a well with reaction solution (cells, CCK-8 solution, and sample solution), cells and CCK-8 solution (without a sample solution), and medium and CCK-8 solution (without cells and sample). Cell viability was determined in triplicate in independent experiments using 6 wells per concentration. IC_50_ values were calculated using SPSS 19 from CCK-8 assay data. 

#### 2.6.3. Mitochondrial Membrane Potential (∆Ψ_m_) 

The mitochondrial membrane potential of S180 cells was assessed using a JC-10 assay, following instructions described by the manufacturer (Solarbio Science and Technology, Beijing, China). The mitochondrial membrane potential assay kit is used to specifically evaluate the ability of a compound to rapidly disrupt the polarity of the mitochondrial membrane. S180 cells were plated in six-well microplates at a concentration of 10^6^ particle/mL and cultured for 24 h. Different concentrations of PE (0, 160, and 200 μg/mL) were added, respectively. At 24 h, the treated cells were incubated with JC-10 at 37 °C and then washed twice with PBS. 

#### 2.6.4. Caspase-3 and Caspase-9 Activity

The caspase-3/9 activity of the S180 cell lysates was determined by using a caspase-3/9 solarbio colorimetric assay kit according to instructions described by the manufacturer (Solarbio Science and Technology, Beijing, China). S180 cells were treated with different concentrations of PE (0, 160, and 200 μg/mL) for 24 h. The old medium was completely replaced. Cells were lysed with lysis buffer for 15 min after being washed with cold PBS, and they were resuspended in trypsin. Caspase-3/9 activity was quantified, and the absorbance was measured at 405 nm using a microplate spectrophotometer. Compared with the untreated cells, the activity was expressed as the fold of enzyme activity. 

### 2.7. Statistical Analysis

All assays were expressed as the mean value ± standard deviation (SD), and all experiments were repeated three independent times in triplicate form. Statistical analysis was conducted using SPSS 19 software (SPSS, Inc., Chicago, IL, USA). Results were tested for statistical significance by one-way analysis of variance (ANOVA). Statistically significant differences among treatments (*p* < 0.001) were assessed using Duncan’s honestly significant difference (HSD) and multiple range tests (DMRT).

## 3. Results and Discussion

### 3.1. Seasonal Variations in Chemical Composition

#### 3.1.1. Seasonal Variations in SSC, SPC, and FAAC

**Figure 1:**
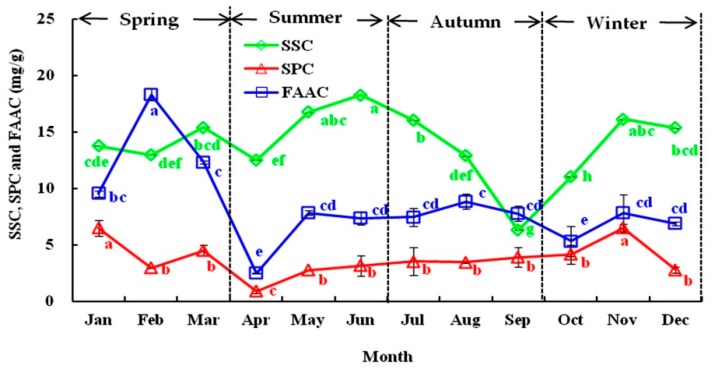
The seasonal variation in the soluble sugar content (SSC), soluble protein content (SPC), and free amino acid content (FAAC) of olive leaves from January to December in 2018. Each value is the mean of triplicate experiments and represent mean ± SD. Values not sharing the same lowercase letters are significantly different (*p* < 0.05).

As shown in Figure 1, the SSC, SPC, and FAAC of the olive leaves varied significantly from January to December, ranging from 6.31 to 18.25, from 0.87 to 6.47, and from 2.49 to 12.30 mg/g dry weight (DW), respectively. The SSC was higher in spring and winter, and the maximal level was detected in May (18.25 mg/g DW) but decreased sharply in autumn. Contrary SSC levels were detected in the study of Eris [35], which reported a continuous increase in SSC and SPC in Gemlik olive leaves grown in autumn and winter. Similarly, the SPC increased in spring and winter, with the maximum content (6.47 mg/g DW) detected in January and November and the lowest content detected in April (0.87 mg/g DW). Starting in June, the SPC remained constant (approximately 3.49 mg/g DW) until October. However, higher quantities of FAAC than SSC and SPC were detected, and the FAAC contents increased moderately during winter and spring, with the maximum value detected in February (18.30 mg/g DW). However, a sharp decline was observed in April (2.49 mg/g DW). Moreover, minimum changes in FAAC were detected from June to September. In general, seasonal variations affected the SSC, SPC, and FAAC values of the olive leaves, which declined in April and September and increased in November. The growth influence of buds, flowers, and fruits may be the most relevant factor and might be positively correlated with the variety [36].

#### 3.1.2. Seasonal Variations in TPC and TFC

**Figure 2:**
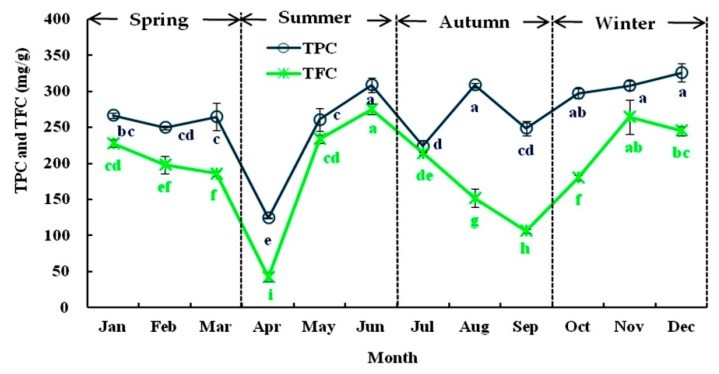
The seasonal variation in the total phenolic content (TPC) and total flavonoid content (TFC) of olive leaves from January to December in 2018. Each value is the mean of triplicate experiments and represent mean ± SD. Values not sharing the same lowercase letters are significantly different (*p* < 0.05).

As shown in Figure 2, the level of phenolics in Liangshan olive leaves varied extensively with the season. TPC and TFC exhibited fluctuations from 124.30 to 325.81 mg GAE/g in January and from 275.32 to 42.20 mg RE/g in December. The maximum levels of TPC and TFC (about 312.00 mg GAE/g and 255.74 mg RE/g, respectively) were detected in May, November, and December, whereas minimal levels were found in April (124.30 mg GAE/g, and 42.30 mg RE/g, respectively) each year. This result was contrary to the study of Papoti et al. [37] who observed an increase in TPC in “Picual” olive leaves from August to November. The changes in TPC may be correlated with the augmentation of the polyphenol protein oxidase content and activity in olive leaves, which is consistent with the finding described by Talhaoui et al. [22]. This suggests that winter may be the best period to obtain phenolic compounds from Liangshan olive leaves, as reported previously. In general, the variation in TPC and TFC content was larger in summer and autumn than in spring and winter. Moreover, an increase in TFC in spring and winter has also been observed by Heimler et al. [38], which could be related with the large increase in biological activities during the renewal of the nutrient cycle of olive leaves [19].

#### 3.1.3. Seasonal Variations in Oleuropein (OE) and Flavonoid Composition

**Figure 3:**
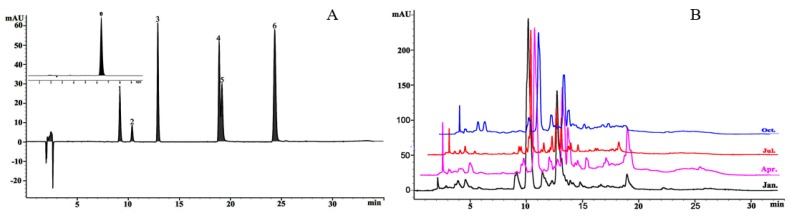
High-performance liquid chromatography (HPLC) chromatograms of phenolic compounds. (**A**) Standard mixture; 0, oleuropein; 1, rutin; 2, luteolin-4’-O-glucoside; 3, apigenin-7-O-glucoside; 4, luteolin; 5, quercetin; 6, apigenin. (**B**) Olive leaf samples in a typical month in 2018.

The HPLC chromatogram of the standard mixture is presented in Figure 3A, and olive leaf samples from January to December were analyzed (Figure 3B). Oleuropein, rutin, luteolin, apigenin, luteolin-4’-O-glucoside, quercetin, and apigenin-7-O-glucoside were identified by comparison to standards (Figure 3A). The chemical composition of the olive leaves was investigated by measuring the seven compounds according to the linear calibration range along with the corresponding calibration equations, retention times, and regression coefficients (Table 1).

Oleuropein was the major phenolic component in Liangshan olive leaves (Table 2), and the levels were consistent with the findings of Orak [39], ranging from 34.45 to 151.74 mg/g DW between January and December. The concentration of oleuropein was substantially elevated in spring and winter, with the maximum value observed in March and the lowest amount detected in June and September. Similar findings were reported by Malik et al. [40]. The seasonal tendency was significantly greater in the cold season than in the warm season, which is consistent with that of Hashemi et al. [41].

Compared with the high levels of OE, smaller amounts of rutin and apigenin-7-O-glucoside were tested, with the maximum concentrations occurring in November (0.91 and 2.47 mg/g DW, respectively), whereas rutin was undetected in April. In contrast, higher amounts of luteolin-4’-O-glucoside were found and ranged from 17.21 to 74.32 mg/g DW between January and December, excluding April, with no significant differences detected during summer and autumn. These results may be explained by the embryonic development in April following the dormancy of the olive trees under the Liangshan climate [24] and likely linked to the increased activity of l-phenylalanine ammonia lyase (PAL) in the phenylpropanoid pathway [42]. Nevertheless, an opposite trend was observed in luteolin, which exhibited a persistent reduction during spring and winter, with the highest values detected in April (Table 2). In addition, quercetin was more concentrated in autumn than in summer, with the maximal level occurring in September. Interestingly, apigenin was only detected in April, May, and August but not in spring or winter. Despite the rarity of this compound in the olive leaves, it was previously notified by Quirantes-Piné et al. [43]. Accordingly, the level of these compounds in the olive leaves was drastically influenced by the floral and fruit developmental stages [40], which might be associated with the chemical and enzymatic reactions that occurred during the period of fruit maturation or as a result of olive processing.

### 3.2. Antioxidant Activity

Based on the seasonal changes results, the chemical composition of the Liangshan olive leaves was higher and relatively stable in January. Therefore, the free radical scavenging ability was evaluated using the phenolic extracts (PEs) derived from Liangshan olive leaves in January. 

#### 3.2.1. DPPH Radical-Scavenging Activity

The DPPH radical-scavenging activity is widely used to estimate the antioxidant capacity of plant extracts. The scavenging ability of PE was analyzed in the concentrations range of 0.2–1.2 mg/mL, and the result was displayed in Table 3. At 1.20 mg/mL, the scavenging effects of PE elicited an appropriate concentration-dependent scavenging effect compared with ascorbic acid (Vc). PE reduced DPPH presenting very low IC_50_ values, whereas the IC_50_ of PE was 0.14 mg/mL (Table 3), suggesting that PE has similar antioxidant capacity to V_C_. The effect may be attributed to the high content of phenolic compounds in PE and the close correlation between phenolic compounds and free radical scavenging of DPPH, with a significantly high correlation coefficient matching that was reported previously in olive leaves [22]. Our results suggest that the DPPH scavenging ability has a direct link between the presence of other compounds in PE and the IC_50_ values. 

#### 3.2.2. Superoxide Radical-Scavenging Activity (O_2_^−^)

Superoxide anion (O_2_^−^) is one of the strongest reactive oxygen species among the free radicals that cause cell injury [44]. The dose–response results were found for PE in the superoxide radical-scavenging assay presented in Table 3. At 1.20 mg/mL, the scavenging rates for PE and Vc were 62.26% and 95.21%, respectively, indicating that PE was lower than Vc. Meanwhile, the higher IC_50_ of PE (0.93 mg/mL) suggested a weaker scavenging capacity (Table 3). The results suggested that a few phenolic antioxidants of PE triggered autoxidation [45], and the phenoxy radical interacted with the oxygen-generating superoxide anion (O_2_^−^) [46,47].

#### 3.2.3. Reducing Power

Antioxidant levels may play a role in determining the reducing power and may be mediated via a reduction in hydrogen capacity. A reduction in Fe^3+^ levels may be correlated with the hydrogen-reducing capacity. It is one of the key antioxidant mechanisms of phenolic compounds. Table 3 presents the results of the reducing power of PE, suggesting a dose–effect relationship between sample concentration and absorbance. At a concentration of 1.20 mg/mL, the values of absorbance of PE and V_C_ were 1.34 and 1.69, respectively, indicating that the reducing power of PE was weaker than V_C_. Szewczyk et al. [48] emphasized that the reducing capacity is linked with reduction, which stabilizes free radicals. Our result indicated that the similar reduction by PE may be attributed to their similar electron-donating or hydrogen-donating capacities.

### 3.3. Anticancer Activity

#### 3.3.1. Effect of PE on Cell Viability in HEK293 Cells

**Figure 4:**
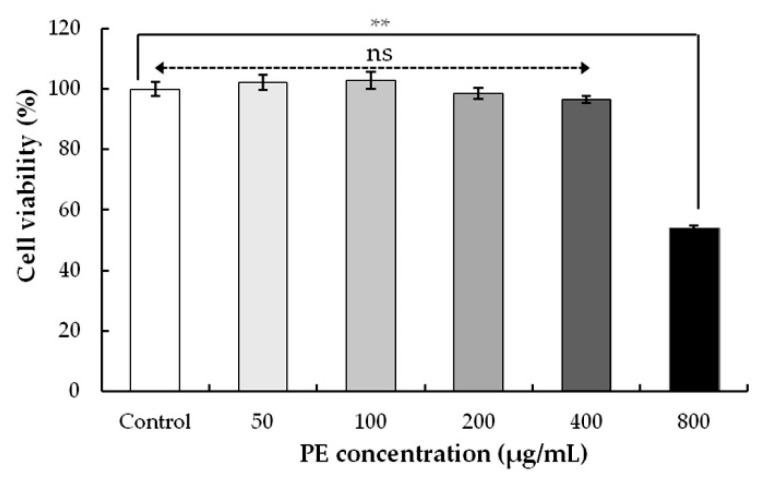
Effect on the cell viability of PE-treated HEK293 cells. Cells were treated with different concentrations (0–800 μg/mL) for 24 h. The cell viability was determined by a CCK-8 assay. Values represent the mean ± SD for three independent experiments (n = 3); ** *p* < 0.01, ns, not significant, refers to the control.

To study the effect of PE on cell viability, HEK293 cells were exposed to PE at 50, 100, 200, 400, and 800 μg/mL for 24 h, and cell viability was assessed by a CCK-8 assay (Figure 4). As compared with the control, no differences in cell viabilities were evaluated when the cells were exposed with 50–400 μg/mL PE. However, when the PE was increased from 400 to 800 μg/mL, PE resulted in a significant cell death (cytotoxicity) of HEK293. The IC_50_ value of HEK293 was found as 841.48 μg/mL (Table 4), indicating the potent toxicity of PE to some concentration range. Similar cytotoxicity against JIMT-1 cells (breast carcinoma) showed that the CC_50_ values (50% cytotoxic concentration) of olive leaf extracts above 800 μg/mL were considered to be ineffective [49]. The cytotoxic activity of olive leaf derived compounds against different cancers (colon, leukemia, and breast cancers) have been demonstrated [50,51,52]. This activity may be due to a special class of phenolic compounds in the extracts, but this requires further study. 

#### 3.3.2. Effect of PE on the Inhibition Rate in HeLa and S180 Cells

**Figure 5:**
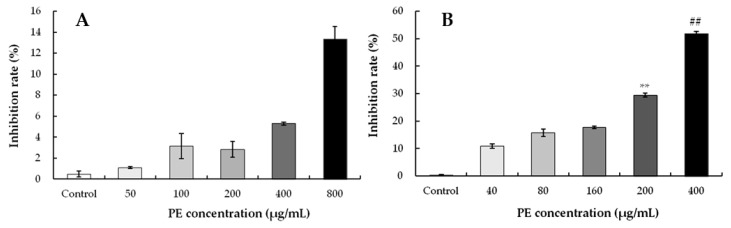
Effect on inhibition rate of PE-treated cells. Cells were treated with different concentrations for 24 h, and the cell viability was detected by a CCK-8 assay. Results represent the mean ± SD for three independent experiments (n = 3). (**A**) The inhibition rate of Hela cells. (**B**) The inhibition rate of S180 cells. ** *p* < 0.01, refers to the control; ## *p* < 0.01, refers to the PE-treated cells of 200 μg/mL.

To find out the appropriate concentration of PE, the cell growth of HeLa cells was assessed via a CCK-8 assay. When PE was in the concentration range of 0 to 800 μg/mL to treat HeLa cells for 24 h, a low inhibition effect of PE was observed (Figure 5A). Further, treatment with 800 μg/mL of PE inhibited cells only by 13.37%, and the high IC_50_ (7139.23 μg/mL) showed that PE at 0–800 μg/mL had no inhibitory effect on HeLa cells (Table 4).

To identify the inhibitory effect of PE, S180 cells were exposed to 0, 40, 80, 160, 200, and 400 μg/mL of PE, and the inhibition rates were determined. As shown in Figure 5B, PE treatment significantly inhibited S180 cell proliferation in a dose-dependent manner. Meanwhile, the IC_50_ value of S180 cells was 457.69 μg/mL with PE treatment for 24 h (Table 4). These results suggested that S180 cells exhibited a higher sensitivity to PE concentrations than HeLa cells. The anti-proliferative activity was consistent with the results reported by Fares et al. [53], who demonstrated that olive leaf extracts exerted anti-proliferative and pro-apoptotic effects in Jurkat cells. In the present work, PE exhibited similar inhibition of S180 cell proliferation, and the inhibition ratios of S180 cells at 200 and 400 μg/mL were relatively high. The effect on the viability of S180 cells suggests that the other compounds in PE exhibit a synergistic effect or induced cellular apoptosis [54].

#### 3.3.3. Treatment with PE Induces Mitochondrial Membrane Potential (∆Ψ_m_) Increase in S180 Cells

**Figure 6:**
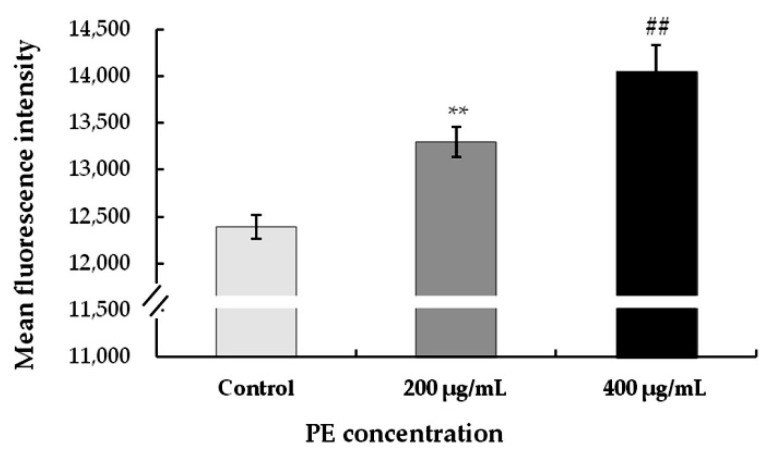
Effect of PE on mitochondrial membrane potential (∆Ψ_m_). S180 cells were treated with different concentrations (0, 200, and 400 μg/mL) for 24 h. The fluorescence intensity for mitochondrial membrane potential in S180 cells was detected by a JC-10 assay according to the kit instructions. Values represent the mean ± SD for three independent experiments (n = 3); ** *p* < 0.01, refers to the control; ## *p* < 0.01, refers to the PE-treated cells of 200 μg/mL.

To evaluate the effect of morphological changes in S180 cells on the treatment with PE, changes in mitochondrial membrane potential are key indicators of cell health, and mitochondrial permeability transition plays an important role in inducing apoptosis. When the mitochondrial membrane potential was reduced, JC-10 could not accumulate in the matrix of mitochondria, in which case JC-10 was a monomer, producing a high intensity of red fluorescence. As shown in Figure 6, compared with the control, the fluorescence intensity was observed with much stronger intensity in S180 cells at concentrations of 200 and 400 μg/mL, indicating a much lower mitochondrial membrane potential. These suggested that PE induced cell mortality by increasing mitochondrial membrane potential depolarization in S180 cells. The result was in agreement with the results of Mijatovic et al. [55], who demonstrated that the extract induced apoptosis of mouse melanoma cells mainly via breakdown of cell membrane integrity. In addition, mitochondria played a crucial step in the induction of cellular apoptosis, and PE-induced cellular apoptosis was associated with mitochondrial membrane potential collapse. One effect of PE was a modulation on mitochondrial membrane potential in S180 cells, indicating the implication of the mitochondrial pathway in S180 cell death induced by this regimen.

#### 3.3.4. PE Induces Caspase-3 and Caspase-9 Activation in S180 Cells

**Figure 7:**
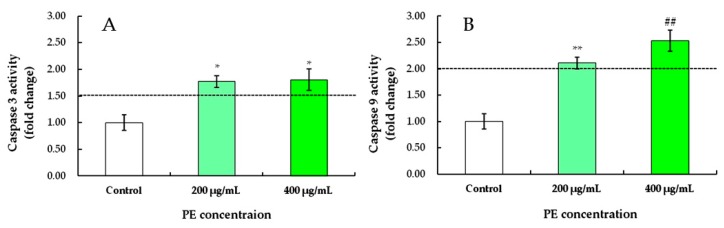
Effects of PE on the activities of caspase-3 (**A**) and caspase-9 (**B**) of S180 cells at concentrations of 0, 200, and 400 μg/mL for 24 h. Values represent the mean ± SD for three independent experiments. * *p* < 0.05, ** *p* < 0.01, refers to the control; ## *p* < 0.01, refers to the PE-treated cells of 200 μg/mL.

To demonstrate the role of mitochondrial pathways in PE-induced apoptosis, the expression levels of proteins related to apoptosis were investigated. After treatment with 200 and 400 μg/mL of PE for 24 h, a marked increase in caspase-3 activities of S180 cells was observed at 1.77- and 1.81-fold higher than that of the control, respectively, but no difference was found between 200 and 400 μg/mL (Figure 7A). 

Similarly, the expression levels of caspase-9 in S180 cells treated with 200 and 400 μg/mL PE were 2.11 and 2.54 times higher that of the control group, respectively (Figure 7B). The higher the PE concentration, the higher the activity of caspase-9 in S180 cells. Caspase-9 is a critical upstream activator of the caspase cascade in vivo, and active site mutations of caspase-9 show a dominant, negative effect on the activation of caspase-3 in transected cells [56]. After treatment with 400 μg/mL of PE, the highest activities of caspase-9 in S180 cells suggested that activated caspase-9 may directly induce specific caspase-activated deoxyribonucleases [57]. These data indicated that PE induced apoptosis of S180 cells by activating caspase-3 and caspase-9 to stimulate mitochondrial cell death [45] via autocatalysis or another mechanism under investigation.

## 4. Conclusions

Olive trees (*Olea europaea* L.) have been cultivated in China for decades, but their variations in chemical composition have not been extensively explored. The objectives of this study were to determine the seasonal variations in the chemical compositions of Liangshan olive leaves from January to December and to evaluate their antioxidant and anticancer activities based on polyphenol content. The results showed that the TPC, TFC, SPC, SSC, and FAAC in Liangshan olive leaves decreased in April and September but increased in spring and winter. HPLC analysis revealed that OE and luteolin-4’-O-glucoside were the primary components, followed by apigenin-7-O-glucoside, quercetin, and rutin, and lower amounts of luteolin and apigenin were detected. These compounds were highest in spring and winter and lowest in summer and autumn as a whole. In addition, excellent antioxidant activity of PE was exhibited when DPPH radical and superoxide radical scavenging were tested, which were 91.29% and 62.26%, respectively, with IC_50_ values of 0.14 and 0.93 mg/mL. The reducing power of PE was also tested. Furthermore, the potent toxicity against HEK293 cells showed that the IC_50_ value of PE was found as 841.48 μg/mL, and a low inhibitory effect was observed when HeLa cells were treated with 0–800 μg/mL of PE for 24 h, but S180 cells exhibited a higher sensitivity to PE concentrations. PE induced S180 cell mortality via increased mitochondrial membrane potential depolarization and up-regulated caspase-3 and caspase-9 activity. Taken together, the potential antioxidant and anticancer activities of phenolic extracts, especially that of Liangshan olive leaves in January and December, can be a potential and alternative source applied in the food, cosmetic, and pharmaceutical fields.

## Figures and Tables

**Table 1:** Analysis of phenolic compounds derived from Liangshan olive leaves *.

CP	LCR (mg/mL)	CE	RC (R^2^)	RT (min)
Oleuropein	0.1–1.7	*y* = 607.01*x* + 9.2935	0.9990	6.388
Rutin	0.1–2.5	*y* = 576.09*x* + 61.592	0.9988	9.166
Luteolin-4’-O-glucoside	1–19	*y* = 336.79*x* + 220.01	0.9902	10.360
Apigenin-7-O-glucoside	0.1–1.3	*y* = 3304.4*x* − 8.3598	0.9995	12.882
Luteolin	0.01–0.031	*y* = 25,711*x* − 17.17	0.9905	18.890
Quercetin	0.005–0.041	*y* = 25,590*x* − 6.9509	0.9991	19.162
Apigenin	0.007–0.021	*y* = 4241.7*x* − 3.6054	0.9944	24.332

* *x*: concentration (mg/mL); *y*: peak area. CP, compound; LCR, linear calibration range; CE, calibration equation; RC, regression coefficient; RT, retention time.

**Table 2:** The seasonal changes in phenolic compounds in olive leaves from January to December (results are presented as mean ± SD expressed as mg of analyte/g of dry matter) *.

Month	Oleuropein	Rutin	Luteolin-4’-O-Glucoside	Apigenin-7-O-Glucoside	Luteolin	Quercetin	Apigenin
**January**	108.57 ± 2.43^c^	0.38 ± 0.043^c^	39.72 ± 2.37^c^	2.07 ± 0.11^ab^	0.057 ± 0.002^cd^	0.463 ± 0.028^b^	nd
**February**	136.70 ± 1.57^b^	0.42 ± 0.038^bc^	63.08 ± 6.14^b^	2.47 ± 0.22^a^	0.061 ± 0.008^cd^	0.537 ± 0.092^b^	nd
**March**	151.74 ± 2.20^a^	0.07 ± 0.012^ef^	17.21 ± 0.79^d^	0.86 ± 0.13^e^	0.093 ± 0.008^cd^	0.458 ± 0.058^b^	nd
**April**	64.55 ± 3.79^f^	nq	nq	0.65 ± 0.42^e^	0.786 ± 0.096^a^	nq	0.177 ± 0.016
**May**	34.45 ± 0.16^h^	0.07 ± 0.006^ef^	11.81 ± 1.41^d^	0.84 ± 0.09^e^	0.365 ± 0.147^b^	nq	nd
**June**	78.23 ± 0.12^e^	0.21 ± 0.003^d^	20.14 ± 0.26^d^	1.47 ± 0.03^cd^	0.149 ± 0.022^c^	0.402 ± 0.066^b^	0.131 ± 0.004
**July**	54.34 ± 1.46^g^	0.13 ± 0.012^de^	10.65 ± 1.41^d^	0.83 ± 0.13^e^	0.044 ± 0.004^cd^	0.164 ± 0.101^c^	nd
**August**	52.16 ± 0.33^g^	0.14 ± 0.012^de^	16.23 ± 1.25^d^	1.02 ± 0.07^de^	0.017 ± 0.001^cd^	0.803 ± 0.080^a^	0.135 ± 0.018
**September**	35.44 ± 0.10^h^	0.34 ± 0.035^c^	16.19 ± 1.34^d^	0.95 ± 0.07^e^	nq	0.462 ± 0.081^b^	nd
**October**	91.42 ± 0.47^d^	0.50 ± 0.062^b^	18.37 ± 2.46^d^	0.61 ± 0.08^e^	0.032 ± 0.007^cd^	0.020 ± 0.001^c^	nd
**November**	142.56 ± 6.44^b^	0.91 ± 0.095^a^	74.32 ± 8.52^a^	1.91 ± 0.19^bc^	0.068 ± 0.002^cd^	0.189 ± 0.094^c^	nd
**December**	106.38 ± 1.47^c^	0.53 ± 0.015^b^	53.81 ± 1.75^b^	1.86 ± 0.05^bc^	0.067 ± 0.001^cd^	0.581 ± 0.009^b^	nd

* Values are presented as mean ± SD of triplicate determinations. The data marked by different letters in a column indicate a significant difference (*p* < 0.05). nd, not detected; nq: not quantified (under the limit of quantification).

**Table 3:** Antioxidant activities of different concentrations of PE from Liangshan olive leaves. PE, phenolic extracts *.

Concentration (mg/mL)		0.20	0.40	0.60	0.80	1.00	1.20	IC_50_
DPPH radical scavenging (%)	VC	92.61 + 0.18 ^b^	93.84 + 0.11 ^a^	93.91 + 0.54 ^a^	93.82 + 0.12 ^a^	94.20 + 0.07 ^a^	94.19 + 0.14 ^a^	
	PE	56.88 + 0.57 ^d^	89.62 + 0.77 ^c^	91.53 + 0.34 ^b^	93.68 + 0.15 ^a^	91.17 + 0.31 ^b^	91.29 + 0.29 ^b^	0.14
Superoxide radical scavenging (%)	VC	95.55 + 0.43 ^a^	95.21 + 0.30 ^a^	95.84 + 0.19 ^a^	95.28 + 0.28 ^a^	95.55 + 0.43 ^a^	95.21 + 0.29 ^a^	
	PE	23.12 + 1.83 ^e^	24.65 + 0.65 ^e^	34.26 + 0.53 ^d^	43.34 + 0.51 ^c^	53.85 + 0.29 ^b^	62.26 + 1.76 ^a^	0.93
Reducing power	VC	1.16 + 0.19 ^b^	1.52 + 0.03 ^a^	1.50 + 0.01 ^a^	1.47 + 0.02 ^a^	1.51 + 0.02 ^a^	1.69 + 0.02^a^	
	PE	0.30 + 0.012 ^f^	0.55 + 0.006 ^e^	0.69 + 0.012 ^d^	0.90 + 0.030 ^c^	1.07 + 0.058 ^b^	1.34 + 0.003 ^a^	

* Each value is the mean ± SD of triplicate independent experiments. The data marked by different letters in a row indicate a significant difference. Values with different superscripts indicate statistical significance by one-way ANOVA and Duncan’s multiple range tests (*p* < 0.05).

**Table 4:** The IC_50_ of PE in cell viability assays.

Cells	IC_50_ (μg/mL)
HEK293	841.48
Hela	7139.23
S180	457.69
